# Relationship between tooth extraction and development of medication-related osteonecrosis of the jaw in cancer patients

**DOI:** 10.1038/s41598-021-96480-8

**Published:** 2021-08-26

**Authors:** Sakiko Soutome, Mitsunobu Otsuru, Saki Hayashida, Maho Murata, Souichi Yanamoto, Shunsuke Sawada, Yuka Kojima, Madoka Funahara, Hiroshi Iwai, Masahiro Umeda, Toshiyuki Saito

**Affiliations:** 1 Department of Oral Health, Nagasaki University Graduate School of Biomedical Sciences, 1-7-1 Sakamoto, Nagasaki, 852-8588 Japan; 2 Department of Clinical Oral Oncology, Nagasaki University Graduate School of Biomedical Sciences, Nagasaki, Japan; 3 Department of Dentistry and Oral Surgery, Kansai Medical University, Osaka, Japan; 4 School of Oral Health Sciences, Kyushu Dental University, Fukuoka, Japan; 5 Department of Otolaryngology, Head and Neck Surgery, Kansai Medical University, Osaka, Japan

**Keywords:** Health care, Oncology

## Abstract

Tooth extraction has been avoided since it has been considered a major risk factor for medication-related osteonecrosis of the jaw (MRONJ). However, MRONJ may also develop from tooth that is an infection source. This study aimed to clarify whether tooth extraction is a risk factor for the development of MRONJ in cancer patients receiving bone-modifying agents (BMAs). This retrospective observational study included 189 patients (361 jaws) from two hospitals. The risk factors of MRONJ were identified by comparing patient characteristics between those who did and did not develop MRONJ. Furthermore, the effect of tooth extraction during BMA therapy was analyzed after adjusting for confounding factors using the propensity score matching method. MRONJ occurred in 33 patients jaws. A longer duration of BMA administration, fewer number of teeth, presence of symptoms of local infection, and infected teeth were independent risk factors of MRONJ. However, tooth extraction during BMA therapy did not increase the risk. Propensity score matching analysis showed that tooth extraction significantly lowered the risk of MRONJ development. Teeth that can be an infection source increases the risk of MRONJ, and thus, they need to be extracted even during BMA administration.

## Introduction

Bone-modifying agents (BMAs) such as zoledronic acid and denosumab are widely used at a high dose for the treatment of skeletal-related events in cancer patients with bone metastases or multiple myeloma. However, patients receiving these drugs have been reported to develop a serious adverse event of medication-related osteonecrosis of the jaw (MRONJ)^1^. MRONJ is a rare mandibular necrosis in patients receiving BMA therapy that mainly presents as bone exposure of the mandible to the oral cavity or skin. Symptoms such as swelling, pain, fistula formation, and pus discharge in the oral cavity or skin are observed, and as they progress, they may cause pathological fractures and sepsis. Although the frequency of MRONJ cannot be clarified because there are few prospective studies, in a phase III study investigating the efficacy and safety of zoledronic acid and denosumab, Saad et al. reported that MRONJ occurred in 89 of 5723 (1.6%) patients^2^. Appropriate first-line treatment of MRONJ is still controversial between conservative and surgical treatments^1,3^.

Studies have shown that invasive dental procedures such as tooth extraction are a risk factor for the onset of MRONJ^4–7^, and therefore, tooth extraction tends to be avoided in these patients. The 2014 American Association of Oral and Maxillofacial Surgeons (AAOMS) position paper on medication-related osteonecrosis of the jaw update described that dentoalveolar surgeries such as tooth extraction are considered a major risk factor for developing MRONJ^1^. The Multinational Association of Supportive Care in Cancer guidelines also recommend that elective dentoalveolar surgical procedures (e.g., nonmedically necessary extractions, alveoloplasties, and implants) should not be performed during active therapy with a BMA at an oncologic dose^8^.

However, avoiding tooth extraction will also preserve the teeth that can be the source of infection, and odontogenic infection is associated with a risk of developing MRONJ. Otto et al.^9^ reported that tooth extraction in patients receiving bisphosphonates can be performed in a safe and predictable way and that the prevailing infectious conditions, and not the tooth extraction itself, may be a key risk factor for the development of MRONJ. We also previously reported that a longer duration of medication and the presence of a tooth with symptoms of local infection were significantly correlated with the development of MRONJ. However, tooth extraction itself was not a risk factor, although no clear conclusion can be drawn because of the small number of patients^10^. Thus, this study aimed to clarify whether tooth extraction is a risk factor for the development of MRONJ in cancer patients receiving BMA and propose an appropriate method of dental intervention in these patients.

## Results

### Characteristics of the evaluated jaws

The average patient age was 62.0 years. Of the 361 jaws analyzed, 215 and 146 jaws were from female and male patients, respectively, and 177 and 184 were the upper and lower jaws, respectively. The characteristics of the 361 jaws are summarized in Table 1. The sort of BMA was bisphosphonate (zoledronic acid) 161 cases, denosumab in 189, and the both in 11. The choice between BP and denosumab was at the discretion of each medical doctor, but denosumab has been used more frequently these days than zoledronic acid. Clinical symptoms of local infection were observed in 30 jaws, whereas teeth that can be a source of infection (periapical lesion larger than 3 mm, alveolar bone loss lager than 1/2, or probing pocket depth larger than 4 mm) were found in 190 jaws.

**Table 1:** Patient characteristics (per jaw).

Variable		Number of jaws/mean ± standard devision	MRONJ (−)	MRONJ (+)
Age (years)		62 ± 11.8	61.6 ± 11.8	65.9 ± 10.7
Sex	Female	215	193	22
	Male	146	135	11
Primary site	Upper jaw	177	164	13
	Lower jaw	184	164	20
Smoking habit	(−)	300	270	30
	(+)	57	54	3
Diabetes	(−)	321	294	29
	(+)	38	34	4
Corticosteroid	(−)	320	290	30
	(+)	41	38	3
Leukocytes (/μL)		2443 ± 2091	2433 ± 2066	2537 ± 2353
Albumin (g/dL)		2.96 ± 0.762	2.95 ± 0.773	3.06 ± 0.641
Sort of BMA	BP	161	149	12
	Dmab	189	169	20
	BP → Dmab	11	10	1
Duration of administration (days)		243 ± 631	221 ± 602	455 ± 850
Number of teeth		11.2 ± 3.58	11.3 ± 3.57	10.5 ± 3.63
Symptom of local infection	(−)	331	311	20
	(+)	30	17	13
Teeth that can be a source of infection	(−)	171	162	9
	(+)	190	166	24
Tooth extraction during BMA therapy	(−)	334	306	28
	(+)	27	22	5

Teeth that can be a source of infection:

periapical lesion (> 3 mm) or alveolar bone loss (> 1/2) or probing pocket depth (> 4 mm).

**Figure 1:**
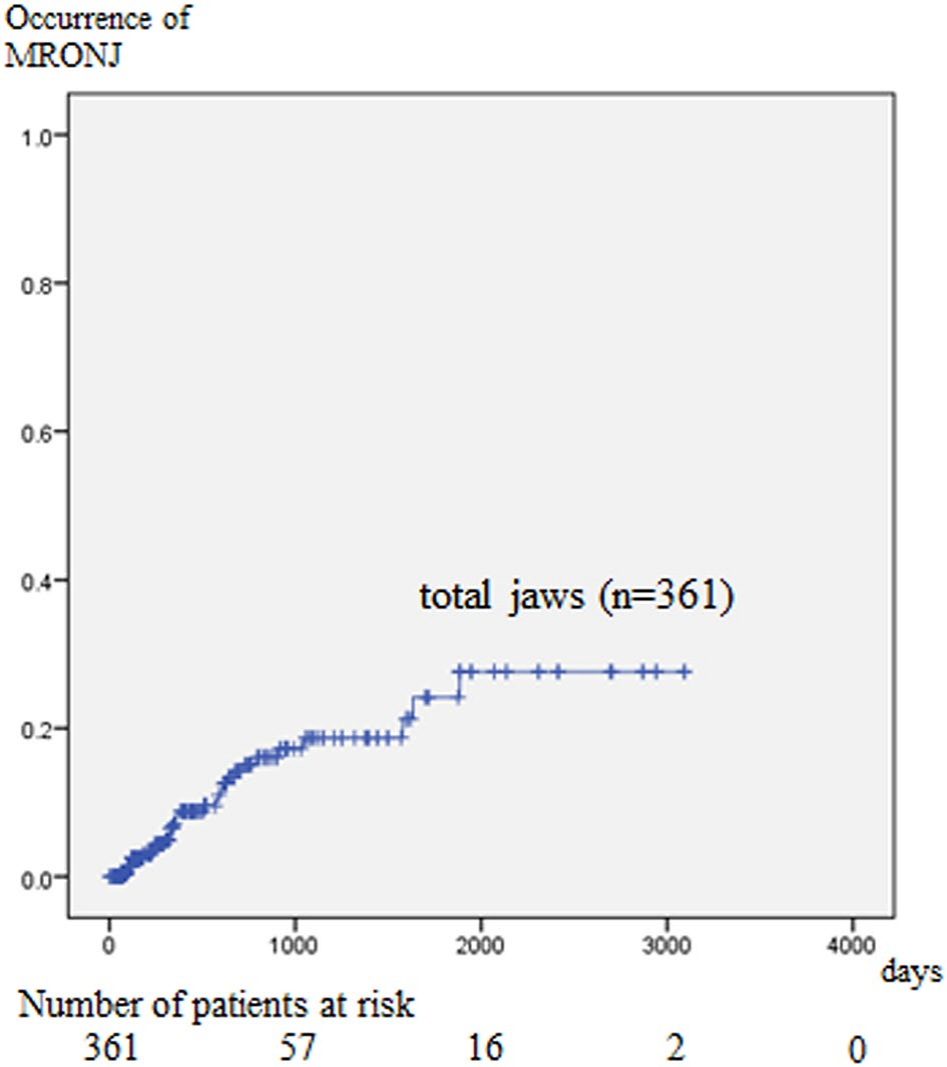
Cumulative occurrence rate of MRONJ. The incidence of MRONJ increases over time, and the 1-, 2-, 3-, 4-, and 5-year cumulative occurrence rates are 8.1%, 14.7%, 18.2%, 18.2%, and 23.3%, respectively.

MRONJ occurred in 33 jaws (13 and 20 upper and lower jaws, respectively). The cumulative incidence rates of MRONJ at 1, 2, 3, 4, and 5 years were 8.1%, 14.7%, 18.2%, 18.2%, and 23.3%, respectively (Fig. 1).

### Risk factors of MRONJ

Table 2 shows the results of the Cox regression analysis for the risk factors of MRONJ. On univariate analysis, the presence of symptoms of local infection (*P* < 0.001) and teeth that can be a source of infection (*P* = 0.006) were significantly correlated with the development of MRONJ. In the Cox proportional hazards model of age, duration of administration of BMA, number of teeth, symptom of local infection, teeth that can be a source of infection, and tooth extraction during BMA therapy, four factors were found to independently influence the risk of MRONJ. These were longer duration of BMA administration (*P* = 0.006), fewer number of teeth (*P* = 0.033), presence of symptom of local infection (*P* < 0.001), and teeth that can be a source of infection (*P* = 0.049). Tooth extraction during BMA therapy did not increase the risk of MRONJ in both univariate and multivariate analyses.

**Table 2:** Relationship between each variable and development of MRONJ (per jaw).

Variable		Univariate analysis			Multivariate analysis		
		*p*-value	HR	95% CI	*p*-value	HR	95% CI
Age (years)		0.083	1.028	0.996–1.060	0.319	1.019	0.983–1.056
Sex	Female vs. male	0.893	1.051	0.509–2.172			
Primary site	upper vs. lower jaw	0.249	1.508	0.750–3.031			
Smoking	(−) vs. (+)	0.302	0.535	0.163–1.756			
Diabetes	(−) vs. (+)	0.739	1.195	0.420–3.402			
Corticosteroid	(−) vs. (+)	0.617	0.738	0.224–2.428			
Leukocytes (10^2^/μL)		0.996	1.000	0.984–1.016			
Albumin (g/dL)		0.379	0.813	0.512–1.290			
Sort of BMA	Bisphosphonate vs. denosumab	0.317	1.351	0.750–2.436			
Duration of administration (months)		0.143	1.008	0.997–1.019	0.010	1.016	1.004–1.029
Number of teeth		0.066	0.920	0.842–1.005	0.033	0.898	0.813–0.991
Symptom of local infection	(−) vs. (+)	< 0.001	8.865	4.397–17.875	< 0.001	7.900	3.605–17.311
Teeth that can be a source of infection	(−) vs. (+)	0.004	3.125	1.450–6.737	0.047	2.381	1.013–5.600
Tooth extraction during BMA therapy	(−) vs. (+)	0.067	2.460	0.940–6.439	0.610	1.298	0.477–3.534

*Cox regression analysis.

Teeth that can be a source of infection:

periapical lesion (> 3 mm) or aloveolar bone loss (> 1/2) or probing pocket depth (> 4 mm).

### Effect of tooth extraction during BMA therapy on the risk of MRONJ

There were significant differences in the patient characteristics between those who did and did not undergo tooth extraction (Table 3). Thus, propensity score matching was performed for an unbiased analysis of the effect of tooth extraction itself on the development of MRONJ. Propensity scores were calculated for 361 jaws using logistic regression analysis of six variables (i.e., age, primary site, duration of administration of BMA, number of teeth, symptom of local infection, and teeth that can be a source of infection). The concordance index (c-index) was 0.557, which indicated a strong capability to differentiate between patients undergoing tooth extraction and not undergoing tooth extraction. The Hosmer–Lemeshow statistic was insignificant (*P* = 0.764), indicating good calibration. The propensity scores, which reflected the probability that a patient would receive tooth extraction during BMA therapy, ranged from 0.01762 to 0.27025 in the non-extraction group, and from 0.01398 to 0.28804 in the extraction group.

**Table 3:** Background factors of patients with and without tooth extraction.

Variable		Without extraction	With extraction	*p* value
Age (years)		61.9 ± 11.7	63.6 ± 12.8	0.463
Primary site	Upper jaw	163	14	0.842
	Lower jaw	171	13	
Duration of administration (days)		229 ± 637	412 ± 534	0.147
Number of teeth		11.3 ± 3.51	9.52 ± 3.96	0.011
Symptom of local infection	(−)	312	19	0.001
	(+)	22	8	
Teeth that can be a source of infection	(-)	166	5	0.002
	(+)	168	22	
Total		334 jaws	27 jaws	

**Figure 2:**
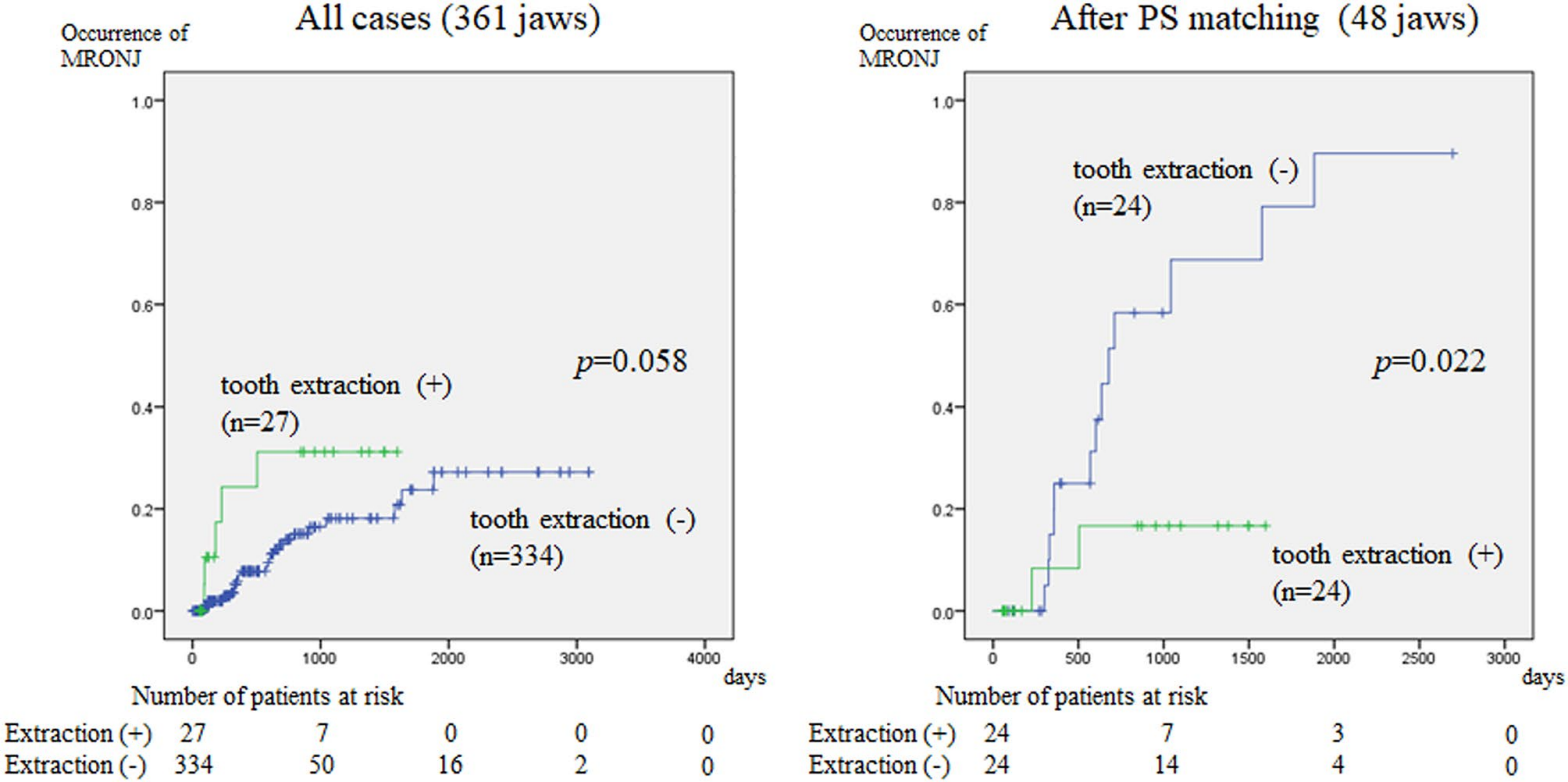
Relationship between tooth extraction and development of MRONJ. (**A**): Overall, MRONJ occurs earlier when tooth extraction is performed, although there is no significant difference in MRONJ incidence between the extraction and no-extraction groups. (**B**). After propensity score matching, the incidence of MRONJ is higher in the no-extraction group, indicating that tooth extraction significantly lowers the risk of MRONJ.

After propensity score matching, the characteristics of the two groups were already balanced (Table 4). The cumulative incidence of MRONJ was calculated using the Kaplan–Meier method for the 48 jaws. Figure 2 shows the relationship between tooth extraction during BMA therapy and the development of MRONJ before and after matching. Before matching, there was no significant difference in the incidence of MRONJ between the tooth extraction group and the no-extraction group. The tooth extraction group tended to develop MRONJ earlier than did the no-extraction group, although the difference in the timing was not significant. After matching, the incidence of MRONJ was significantly lower in the tooth extraction group than that in the no-extraction group.

**Table 4:** Background factors of patients with and without tooth extraction after propensity score matching.

Variable		Without extraction	With extraction	*p* value
Age (years)		66.2 ± 9.44	62.4 ± 13.0	0.259
Primary site	Upper jaw	13	12	1.000
	Lower jaw	11	12	
Duration of administration (days)		506 ± 980	308 ± 408	0.368
Number of teeth		10.9 ± 2.91	9.83 ± 3.92	0.301
Symptom of local infection	(−)	17	19	0.740
	(+)	7	5	
Teeth that can be a source of infection	(−)	7	5	0.740
	(+)	17	19	
Total		24 jaws	24 jaws

## Discussion

In this retrospective observational study, it was revealed that tooth extraction is not a risk factor for developing MRONJ in cancer patients receiving high-dose BMA, but that preserving teeth that require tooth extraction is a risk factor for developing MRONJ.

Many observational studies have reported that tooth extraction is a major risk factor for the development of MRONJ in cancer patients receiving BMA therapy, and thus, tooth extraction has been avoided. Several studies have also reported that MRONJ occurs in 52–61% of patients with a history of tooth extraction. In a longitudinal cohort study by Vahtsevanos et al.^6^, 60/1621 (4.9%) patients with breast cancer, prostate cancer, or multiple myeloma treated with bisphosphonate developed MRONJ. Furthermore, tooth extraction was associated with a 33-fold increase in the risk of MRONJ. Saad et al.^2^ reported that 89 of 5723 patients (1.6%) with bone metastasis secondary to solid tumor or multiple myeloma developed MRONJ, and 61.8% of the MRONJ patients underwent tooth extraction during BMA therapy. Felm et al.^11^ reported that 10 of 345 (2.9%) patients with breast cancer or gynecological malignancies developed MRONJ, and 6 of the 10 MRONJ patients had a history of recent tooth extraction. Kyrgidis et al.^5^ also reported that a history of tooth extraction during zoledronic acid treatment increased the risk of developing MRONJ by 16-fold in 20 MRONJ patients with breast cancer. Similarly, there are many reports that tooth extraction is a risk of developing MRONJ^12–18^.

However, some studies also reported that tooth extraction during BMA therapy does not influence the risk of developing MRONJ. Otto et al.^9^ reported that tooth extraction is safe and feasible in patients receiving bisphosphonates, even in high-risk patients, when performed according to established guidelines. Further, it is not the tooth extraction itself, but rather the prevailing infectious conditions that may be a key risk factor for the development of MRONJ. Similarly, our previous study of 135 cancer patients receiving BMA therapy, 18 of whom developed MRONJ, showed that the risk factors for developing MRONJ were a longer duration of BMA administration and the presence of local infection symptom. However, tooth extraction did not increase such risk^10^. Tooth extraction as a risk factor for developing MRONJ is based on the fact that many MRONJ patients have a history of tooth extraction or that MRONJ commonly occur in BMA-treated patients who undergo tooth extractions. However, most teeth that require extraction are associated with local infections, such as periodontal disease or periapical lesion. Thus, a matched analysis would achieve more accurate results considering the large bias between patients who undergo and do not undergo tooth extraction.

However, to our best knowledge, no study has compared the incidence of MRONJ between extraction and no-extraction cases after adjusting for confounding factors of local infection. In this study, although the difference was not significant, the incidence of MRONJ was higher in the tooth extraction group than in the no-extraction group. However, after adjusting for confounders, the incidence of MRONJ was significantly higher in the no-extraction group. This indicates that extracting teeth with local infection (e.g., periodontal disease, periapical lesion, or deep probing pocket depth) lowers the risk of MRONJ. The Kaplan–Meier curves for the cumulative incidence of MRONJ also showed no difference between the two groups up to after 1 year. Thereafter, the no-extraction group showed a higher onset curve than the extraction group. From a clinical point of view, we think that in patients receiving BMA, teeth that can be a source of infection should be extracted. Especially in patients with a long expected prognosis, tooth extraction is highly recommended because the incidence of MRONJ increases significantly as the survival time increases without tooth extraction. Even if the expected prognosis for life is short, tooth extraction itself does not become a risk of developing MRONJ, so it is not appropriate to hesitate to extract teeth if there are clinical symptoms and conservative dental treatment cannot be expected to cure.

Although tooth extraction should be considered in cancer patients receiving BMA who have teeth that can be a source of infection, it is also known that the incidence of MRONJ after tooth extraction is high. Thus, a tooth extraction method that does not cause MRONJ needs to be developed. In our previous multicenter retrospective analysis of 163 tooth extractions in cancer patients receiving BMA therapy, MRONJ occurred in 25.2% of patients with teeth and root amputation. Immunosuppressive therapy, extraction of mandibular teeth, extraction of teeth with pre-existing inflammation, and a longer duration (≥ 8 months) of high-dose BMA therapy were significantly associated with MRONJ^19^. Otto et al.^9^ performed tooth extraction with the following prophylactic interventions: (1) prolonged antibiotic prophylaxis, (2) atraumatic extraction, (3) smoothing of sharp bony edges, and 4) mucosal wound closure. Only 4 of the 43 cancer patients receiving BMA therapy developed MRONJ. Lodi et al.^20^ advocated the following procedures to prevent MRONJ: (1) mouth rinsing with 0.2% chlorhexidine once a day; (2) removal of dental plaques and calculi and professional oral hygiene treatment 2–3 weeks before the extraction; (3) starting 1 g amoxicillin every 8 h 3 days before the extraction and continuing for 17 days; and (4) debridement and curettage of the extraction socket to remove all granulation and infected tissues, vertical incision in the mesial and distal teeth, coronally advancing the flap, and finally mucoperiosteal flap repair to completely close the extraction socket. None of the 23 cancer patients receiving BMA therapy who underwent tooth extraction according to the above procedures developed MRONJ.

**Figure 3:**
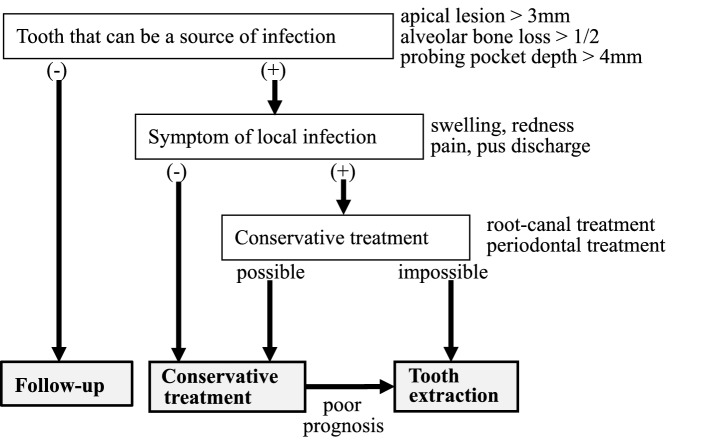
Schema for dental intervention in patients receiving high-dose BMA.

Another study on tooth extraction methods also advocated the effectiveness of primary wound closure and perioperative antibiotic therapy for the prevention of MRONJ after tooth extraction^21,22^. Şahin et al.^23^ reported that platelet-rich fibrin effectively prevents the onset of MRONJ after tooth extraction in both osteoporosis and cancer patients receiving BMA. However, despite the several studies on tooth extraction methods that suppress the onset of MRONJ, most of them have no control group or found no significant difference in the incidence of MRONJ between the treatment and control groups. A systematic review by Lopez-Jornet et al.^24^ concluded that there are no published scientific data to sufficiently support any specific treatment protocol, including the use of autologous platelet concentrate, for the treatment or prevention of MRONJ. The results of our study support the need for dental intervention in patients receiving high-dose BMA (Fig. 3). Apical lesions of 3 mm or more, alveolar bone loss of 1/2 or more, and probing pocket depth of 4 mm or more are considered as teeth that can be infected. Teeth that can be a source of infection with local infection symptoms (swelling, pain, redness, or pus discharge) and are difficult to treat conservatively are extracted. Meanwhile, those without local infection but can still be a source of infection can be treated with conservative dental procedures such as root canal and periodontal treatment including periodontal surgery.

This study had some limitations. First, this was a retrospective observational study and not a controlled study. Although the confounding factors in the extraction and no-extraction groups were adjusted with propensity score matching, it is still desirable to conduct a case–control study because of the possibility of unknown confounding factors. Second, because tooth extraction has been avoided in patients receiving high-dose BMA, only a small number of patients remained after matching. Third, the factors that may affect the healing process, such as tooth extraction procedures and antibiotic administration periods, are yet to be established. Despite these limitations, we believe that this study is valuable because to the best of our knowledge, this is the first study to show via propensity score matching analysis that early tooth extraction lowered the incidence of MRONJ in cancer patients receiving high-dose BMA therapy.

In conclusion, this is the first study to show that in patients with teeth that can be a source of infection, extraction of teeth even after the start of BMA administration may reduce the risk of developing MRONJ. In addition, we attempted to create a flow chart of recommended dental procedures for patients receiving high-dose BMA. We hope that similar research will be conducted in the future and that a method for preventing MRONJ in BMA-administered patients will be established.

## Methods

### Study design and patients

This multicenter, retrospective observational study included 189 patients with malignant tumors who received high-dose BMA therapies (zoledronate 4 mg/4 weeks, or denosumab 120 mg/4 weeks) who first visited Nagasaki University Hospital between January 2011 and December 2019 or Kansai Medical University Hospital between January 2014 and December 2019. Since a certain follow-up period is required, the final observation date was set to June 30, 2020. All patients were referred to the dental unit of the relevant hospital and underwent panoramic X-ray examinations and dental treatments. Patients receiving BMA underwent oral hygiene guidance, removal of dental calculus, professional mechanical tooth cleaning, and other necessary dental treatments. Japanese Position Paper^3^ recommends that tooth extraction be avoided as much as possible in patients receiving high-dose BMA. However, if treatment is difficult other than tooth extraction, the dentist may decide to extract the tooth. Patients were followed up for as long as their general condition allowed, and clinical and imaging examinations were performed every 3 months. Patients who were not followed up for at least 3 months were excluded from the study. Given that there may be a difference in the incidence of MRONJ after tooth extraction between the maxilla and mandible, the evaluation was performed per jaw and not per person. MRONJ was diagnosed using the AAOMS criteria^1^. After excluding 17 edentulous jaws of 17 patients, 361 jaws of 189 patients were evaluated.

### Variables

The variables examined were as follows: (1) demographic factors of age and sex; (2) general conditions: diabetes, smoking habits, administration of corticosteroids, leukocyte count, and serum albumin levels; (3) treatment factors: type of BMA (BP or Dmab) and duration from the start of BMA administration to referral to the dental unit; (4) dental factors: number of residual teeth, clinical symptoms of local infection (pain, swelling, redness, or pus discharge) and abnormal X-ray findings (periapical lesion larger than 3 mm, alveolar bone loss of more than 1/2, probing pocket depth larger than 4 mm, and pericoronitis), and tooth extraction after starting BMA administration; and (5) development of MRONJ. Referring to Yamagata's report^25^ on oral management of hematopoietic stem cell patients, but from a more preventive point of view, tooth with periapical lesions larger than 3 mm, alveolar bone loss of more than 1/2, or probing pocket depth larger than 4 mm was defined as "a tooth that can be a source of infection". Tooth extractions were performed by various dentists rather than by a specific dentist. Japanese Position Paper^3^ recommends removal of bone edge and closure with mucosal periosteal flap when extracting teeth in patients receiving high-dose BMA. Therefore, in all tooth extraction cases, the wound was primary sutured.

### Statistical analysis

The cumulative occurrence rate of MRONJ was calculated using the Kaplan–Meier method and was analyzed using univariate and multivariate Cox regression analysis. To confirm the effect of post-BMA tooth extraction on the risk of MRONJ, the characteristics of the patients who did and did not undergo tooth extraction cases were adjusted using propensity score matching. Propensity scores of tooth extraction and non-extraction groups were calculated using logistic regression analysis, and the propensity score values of both groups were matched with 1/4 of the standard deviation of the propensity score as the allowable value. Then the cumulative incidence rate was calculated with the Kaplan–Meier method and analyzed using the log rank test. All statistical analyses were performed using SPSS software (version 24.0; Japan IBM Co., Tokyo, Japan). All analyses were two-tailed, and P values < 0.05 were considered statistically significant.

### Ethics

The study protocol conformed to the ethical guidelines of the Declaration of Helsinki and the Ethical Guidelines for Medical and Health Research involving Human Subjects by the Ministry of Health, Labor and Welfare of Japan. Ethical approval was obtained from the Institutional Review Boards (IRB) of Nagasaki University Hospital (No. 21021501). Japanese law does not require individual informed consent from participants in non-invasive observational trials such as the present study. Therefore, the need for informed consent was waived. As this was a retrospective study, patient identifiable information was removed, and the research plan was published on the homepages of the participating hospitals websites, along with an opt-out option in accordance with IRB instructions.

## Data Availability

The data that support the findings of this study are available from the corresponding author upon reasonable request.
